# Combined internal fixation and autologous chondrocyte implantation for tibial plateau fracture concomitant with cartilage injury: a case report

**DOI:** 10.1093/jscr/rjaf078

**Published:** 2025-02-21

**Authors:** Yasuhito Sogi, Masahiro Ohnuma, Tomonori Kunii, Atsushi Takahashi, Toshimi Aizawa

**Affiliations:** Department of Orthopaedic Surgery, Japanese Red Cross Sendai Hospital, 2-43-3 Yagiyamahoncho, Taihaku-ku, Sendai 982-8501, Japan; Department of Orthopaedic Surgery, Japanese Red Cross Sendai Hospital, 2-43-3 Yagiyamahoncho, Taihaku-ku, Sendai 982-8501, Japan; Department of Orthopaedic Surgery, Tohoku Rosai Hospital, 4-3-21 Dainohara, Aoba-ku, Sendai 981-8563, Japan; Department of Orthopaedic Surgery, Joint Surgery, Sports Clinic Ishinomaki, 5-2-1 Ayumino, 986-0850 Ishinomaki, Japan; Department of Orthopaedic Surgery, Tohoku University School of Medicine, 1-1 Seiryo-machi, Aoba-ku, Sendai 980-8574, Japan

**Keywords:** tibial plateau fracture, cartilage injury, autologous chondrocyte implantation

## Abstract

This is the first report of tibia plateau fracture (TPF) concomitant with a large cartilage injury, which was treated with consecutive procedures comprising internal fixation and autologous chondrocyte implantation (ACI). A 49-year-old male patient was diagnosed with a medial tibial plateau fracture concomitant with a large medial femoral condyle cartilage injury. The patient underwent internal fixation and ACI at a 4-week interval. One year after the surgery, he could resume daily activities and sports. Magnetic resonance imaging and arthroscopic findings showed that the injured cartilage was covered by hyaline cartilage-like tissue. TPF concomitant with large cartilage injury can be treated with consecutive internal fixation and ACI procedures, shortening the overall treatment period and favorable outcomes.

## Introduction

Tibial plateau fractures (TPF) are complex intra-articular injuries that can lead to post-traumatic osteoarthritis, especially when there is articular displacement [1, 2]. Concomitant soft-tissue injuries, like meniscal tears, ligament injuries, and cartilage injuries, are common and associated with short-term outcomes [1, 3, 4]. While magnetic resonance imaging (MRI) is useful for detecting these injuries preoperatively, it is not routinely performed. There is limited information on treating TPFs with associated cartilage injuries, despite successful autologous chondrocyte implantation (ACI) for isolated cartilage injuries [5]. However, the outcomes of ACI in patients with cartilage injuries accompanied by TPFs are unclear. Herein, we present a case of TPF concomitant with a large cartilage injury treated with consecutive procedures comprising internal fixation and ACI at a 4-week interval.

## Case report

The review board of our institution approved this study, and informed consent was obtained from the patient.

**Figure 1:**
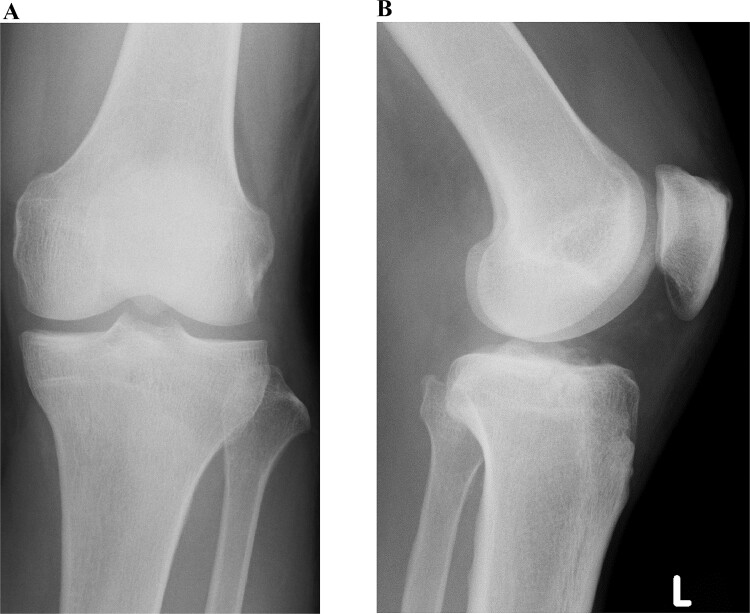
Preoperative plain radiographic images of the left knee. Articular depression of the tibia is shown on a lateral-view radiograph.

**Figure 2:**
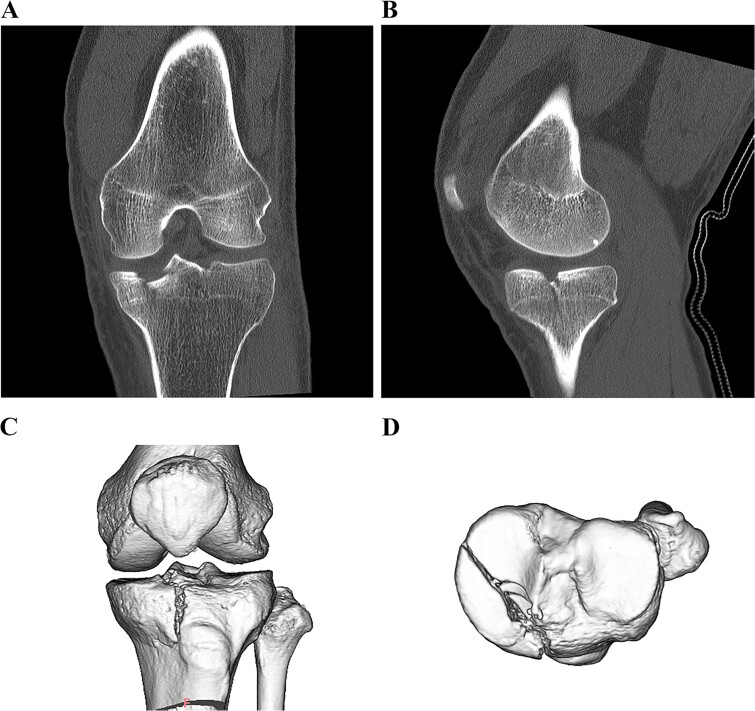
Preoperative CT scan images of the left knee. Coronal and sagittal CT scan images (A, B). 3D CT scan images (C, D). A 5-mm depression in the medial articular surface and column fracture of the tibial plateau are revealed.

**Figure 3:**
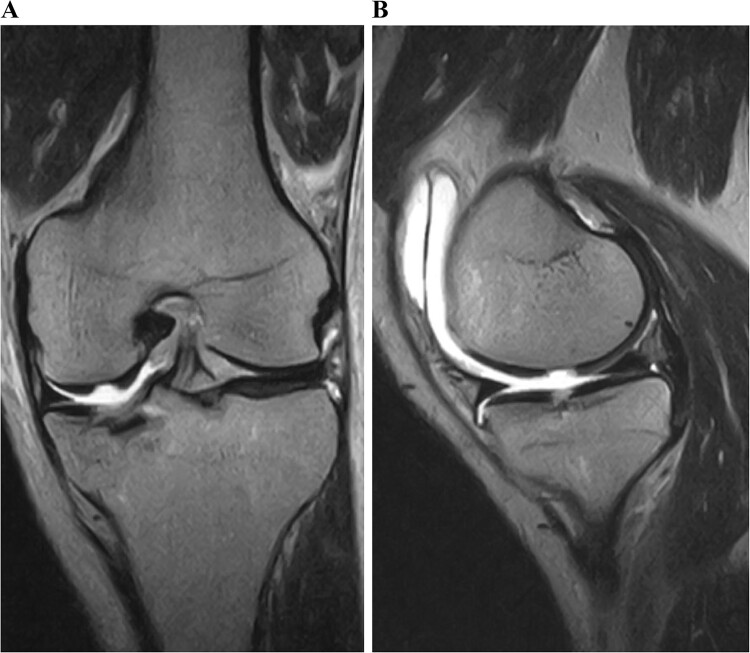
Preoperative T2-weighted magnetic resonance images of the left knee. A cartilage injury of the medial femoral condyle appeared as a kissing lesion opposite the tibial plateau fracture. The coronal image revealed a 20-mm-wide cartilage injury (A). The sagittal image showed an 18-mm-long cartilage injury (B).

A 49-year-old male patient fell while playing soccer and visited our hospital with severe pain and swelling in his left knee. A lateral-view plain radiograph showed articular depression of the tibial plateau (Fig. 1). Three-dimensional reconstructed computed tomography (CT) scan revealed a 5-mm depression of the medial articular surface and a split in the medial plateau (Fig. 2). The fracture was classified as AO/OTA 41B3.2 and Schatzker type 2 [6, 7]. MRI scan showed a medial femoral cartilage injury that appeared as a kissing lesion opposite the tibial fracture. The length and width of the cartilage defect on the femoral condyle were 18 mm and 20 mm, respectively (Fig. 3). A part of the peripheral cartilage around the defect was suspected to be delaminated, and the defect was estimated to exceed 4 cm^2^.

**Figure 4:**
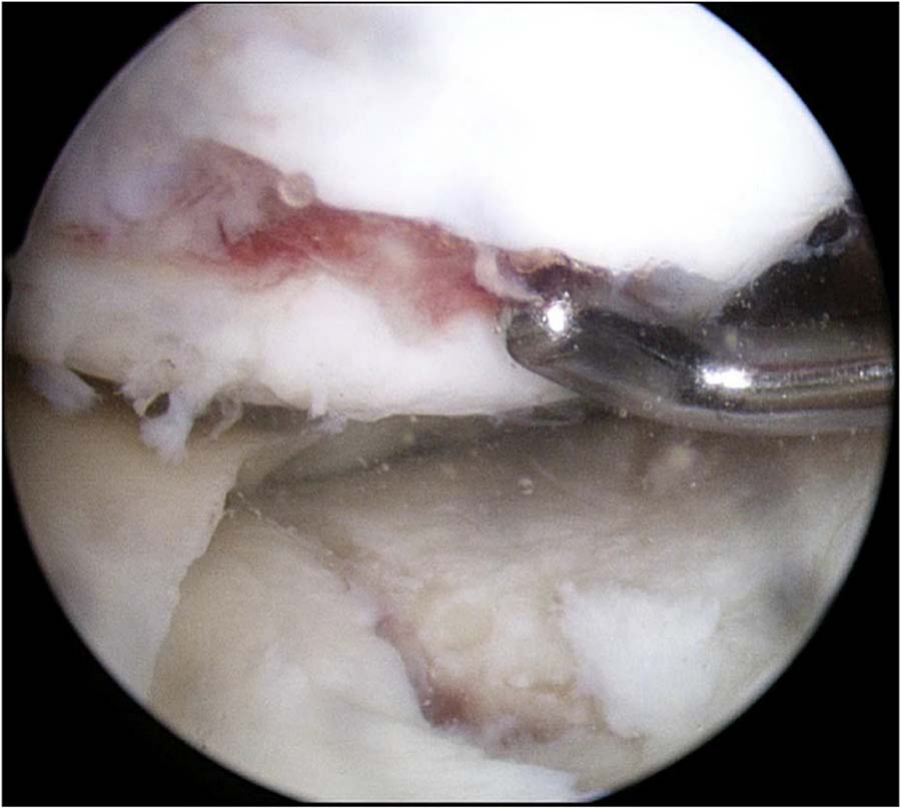
Arthroscopic findings of the medial compartment of the left knee at the initial surgery. Complete cartilage loss of the femoral condyle to the subchondral area and a depressed medial tibial plateau were observed.

**Figure 5:**
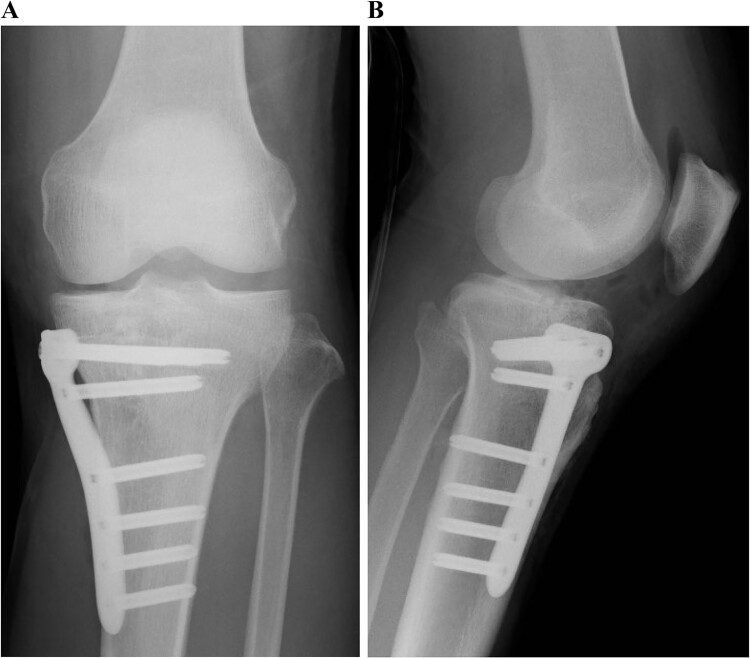
Postoperative plain radiographs of the left knee. Internal fixation was performed with the TriS Medial HTO Plate System.

Consecutive surgeries, including internal fixation for TPF and ACI for cartilage injury, were planned. During the first surgery, the arthroscopic examination revealed ICRS grade IV cartilage injury of the medial femoral condyle and depressed medial tibial plateau (Fig. 4). The depressed medial tibial plateau was elevated and reduced. Then artificial bone chips were inserted into the defect beneath the plateau, and the fracture was fixed using the TriS Medial HTO Plate System (Olympus Terumo Biomaterials Corp., Tokyo, Japan) (Fig. 5). Finally, approximately 0.5 mg of healthy cartilage was harvested from a non-weight-bearing area of the lateral femoral condyle. The cartilage was cultured in a three-dimensional environment at the facility (Japan Tissue Engineering Co., Ltd., Gamagori, Japan) for 4 weeks. Next, open ACI was performed 4 weeks after the initial surgery, as described in a previous study [8]. The cultured cartilage was transplanted into the cartilage defect of the femur and covered by the periosteum, which was harvested from the proximal tibia [9]. The periosteum was sutured around cartilage using the 1.4 mm JuggerKnot® soft anchor system (Zimmer Biomet, Warsaw, IN, USA). Postoperative rehabilitation included partial weight bearing at 4 weeks and full weight bearing at 8 weeks. The patient was permitted to perform heavy labor and sports activities 9 months after the surgery.

**Figure 6:**
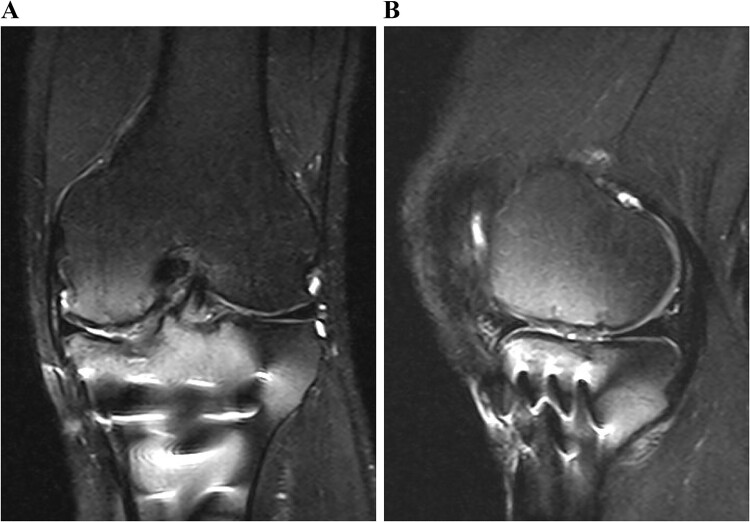
Postoperative T2-weighted fat-suppressed magnetic resonance images at 1 year after surgery. The transplanted cultured cartilage was successfully integrated without any delamination.

**Figure 7:**
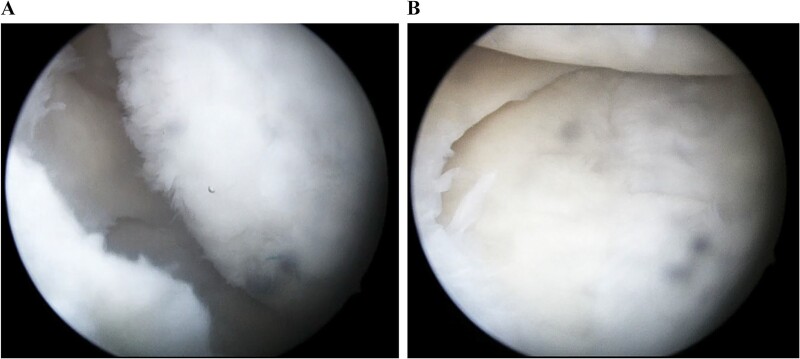
Arthroscopic findings of the medial compartment of the left knee 1 year after the surgery. The cartilage defect of the medial femoral condyle was covered by hyaline cartilage-like tissue (A). The depressed articular surface of the medial tibia plateau was smooth without step-off (B).

Postoperative MRI at 1 year after the surgery showed that the transplanted cultured cartilage had successfully integrated without any delamination (Fig. 6). A second-look arthroscopy was performed to evaluate the condition of the transplanted cartilage while removing the plate. The cartilage defect of the medial femoral condyle was covered by hyaline cartilage-like tissue. The depressed articular surface of the medial tibia plateau was smooth without stepping off (Fig. 7). One year after the surgery, the patient resumed daily life and sports activities without any complaint. The patient’s range of motion of the knee was full, and Lysholm’s knee score was 95.

## Discussion

This is the first report of a case of TPF concomitant with a large cartilage injury detected by preoperative MRI, which was treated with consecutive procedures comprising internal fixation and ACI.

Cartilage injuries can occur simultaneously with TPFs. However, the compartment in which such injuries occur is not well understood. In this case, the injury occurred in the same compartment as the fracture. To the best of our knowledge, this is the first case report of a femoral cartilage injury presenting as a kissing lesion opposite the tibial fracture. Excessive axial or varus and valgus forces are associated with developing TPFs and might cause femoral cartilage injury [10].

It is generally recognized that an irregular articular surface after surgical treatment for TPF has a negative effect on functional outcomes and the progression of radiographic osteoarthritis [11, 12]. However, the impact of concomitant cartilage injuries on outcomes is unclear. There have been only a few reports about long-term outcomes after cartilage injuries. A previous study with a follow-up period of over 19 years showed that ACI had superior patient-reported outcomes to those of untreated injuries, bone marrow stimulation, and osteochondral autograft transplantation [13]. Therefore, if a large cartilage injury associated with TPFs is identified preoperatively, it should be treated as consecutive internal fixation and ACI procedures. Although ACI requires a second-stage surgery, cartilage harvesting can be performed during internal fixation, leading to shortening the overall treatment period.

Depending on the lesion size, cartilage injuries can be treated with bone marrow stimulation, osteochondral autograft transplantation, or ACI. Another study has reported the long-term outcomes of early-generation ACI for isolated cartilage injury [5]. However, the use of ACI for patients with fractures has not been examined. Acute bleeding or joint hemorrhage attributed to fractures can cause cartilage degeneration via hemosiderin synovitis [14]. Therefore, performing ACI for patients with fractures might be inadequate. However, in this case, the arthroscopic findings showed good cartilage healing. Good healing may be attributed to the treatment period of 4 weeks between the initial surgery and chondrocyte implantation or the favorable properties of the harvested cartilage.

Evaluating the condition of the cartilage before surgery is important for planning surgical strategies. Preoperative planning is primarily based on conventional radiographs and CT, which are insufficient for diagnosing soft-tissue and cartilage injuries. MRI is not routinely performed for such cases [15]. A previous report has recommended preoperative MRI for detecting meniscal injury. Further, the current study also suggests a preoperative MRI for identifying cartilage damage.
